# Physical Exercise Training versus Relaxation in Allogeneic stem cell transplantation (PETRA Study) – Rationale and design of a randomized trial to evaluate a yearlong exercise intervention on overall survival and side-effects after allogeneic stem cell transplantation

**DOI:** 10.1186/s12885-015-1631-0

**Published:** 2015-09-07

**Authors:** Joachim Wiskemann, Rea Kuehl, Peter Dreger, Gerhard Huber, Nikolaus Kleindienst, Cornelia M. Ulrich, Martin Bohus

**Affiliations:** 1 National Center for Tumor Diseases (NCT) and Heidelberg University Hospital, Heidelberg, Germany; 2 National Center for Tumor Diseases (NCT) and German Cancer Research Center, Heidelberg, Germany; 3 Central Institute of Mental Health, Mannheim, Germany; 4 Department of Medicine V, Heidelberg University, Heidelberg, Germany; 5 Institute of Sports and Sport Science, Heidelberg University, Heidelberg, Germany; 6 Huntsman Cancer Institute, Salt Lake City, USA; 7 Faculty of Health, University of Antwerp, Antwerp, Belgium

## Abstract

**Background:**

Allogeneic stem cell transplantation (allo-HCT) is associated with high treatment-related mortality and innumerable physical and psychosocial complications and side-effects, such as high fatigue levels, loss of physical performance, infections, graft-versus-host disease (GvHD) and distress. This leads to a reduced quality of life, not only during and after transplantation, but also in the long term. Exercise interventions have been shown to be beneficial in allo-HCT patients. However, to date, no study has focused on long-term effects and survival. Previous exercise studies used ‘usual care’ control groups, leaving it unclear to what extent the observed effects are based on the physical effects of exercise itself, or rather on psychosocial factors such as personal attention. Furthermore, effects of exercise on and severity of GvHD have not been examined so far. We therefore aim to investigate the effects and biological mechanisms of exercise on side-effects, complications and survival in allo-HCT patients during and after transplantation.

**Methods/design:**

The PETRA study is a randomized, controlled intervention trial investigating the effects of a yearlong partly supervised mixed exercise intervention (endurance and resistance exercises, 3–5 times per week) in 256 patients during and after allogeneic stem cell transplantation. Patients in the control group perform progressive muscle relaxation training (Jacobsen method) with the same frequency. Main inclusion criterion is planned allo-HCT. Main exclusion criteria are increased fracture risk, no walking capability or severe cardiorespiratory problems. Primary endpoint is overall survival after two years; secondary endpoints are non-relapse mortality, median survival, patient reported outcomes including cancer related fatigue and quality of life, physical performance, body composition, haematological/immunological reconstitution, inflammatory parameters, severity of complications and side-effects (e.g. GvHD and infections), and cognitive capacity.

**Discussion:**

The PETRA study will contribute to a better understanding of the physiological and psychological effects of exercise training and their biological mechanisms in cancer patients after allo-HCT. The ultimate goal is the implementation of optimized intervention programs to reduce side-effects and improve quality of life and potentially prognosis after allogeneic stem cell transplantation.

**Trial registration:**

ClinicalTrials.gov Identifier: NCT01374399.

## Background

Allogeneic stem cell transplantation (allo-HCT) is the only curative medical treatment option for patients with haematological malignancies in high-risk situations e.g. acute leukaemia. However, patients suffer from numerous treatment related side-effects and complications, and the transplant-related mortality is high [1]. Exercise constitutes a potentially promising intervention approach for this patient group. Over the last years, several clinical trials have contributed to the growing body of evidence showing the beneficial effects of exercise in cancer patients [2–5], and some general exercise recommendations for cancer patients have already been published [6] also in the field of allo-HCT [7].

Our group has reviewed exercise intervention studies in the context of stem cell transplantation and illustrated that exercise interventions at different time points during and after HCT might significantly improve physical performance, quality of life, symptom control and fatigue [7]. Since publication of this review, 6 new randomized controlled trials (RCTs) have been published supporting the findings [8–13]. These studies were included in a recent review and meta-analysis by Persoon et al. [14] and the authors found that exercise significantly improved cardiorespiratory fitness, lower extremity muscle strength and fatigue and had also a small effect on upper extremity muscle strength, quality of life (QoL), physical, emotional and cognitive function. The researcher concluded that more high-quality studies were needed [14]. However, it is still not possible to give patients clear advice regarding the best type, intensity, start and duration of an exercise program.

Prior to allo-HCT, patients’ physical performance is already affected due to the disease itself and/or previous treatment [15, 16]. Furthermore, emerging evidence indicates that cancer patients have considerably impaired cardiorespiratory fitness as a result of the toxic effects of anticancer therapy or as a consequence of the disease (for example cachexia, deconditioning, anaemia) [17]. Thus, physical activity levels have been described as generally low in a group of haematological cancer survivors [18]. Furthermore, one study compared the quality of life of 662 HCT survivors with age- and sex-matched healthy controls and observed poorer general health, physical function, well-being, depression, cognitive function, and fatigue in HCT survivors [19]. A major complication after allo-HCT is graft-versus-host disease (GvHD). GvHD is the leading cause of morbidity and high transplant-related mortality. It is characterized by a reaction of donor T-cells against patient tissues e.g. mucosa or skin [1, 20]. Moreover, chronic GvHD is associated with a lower physical performance and functional capacity [21]. A recent review shows that patients after HCT are likely to have long-term difficulties with physical functioning, problems with fatigue, distress and a deteriorated psychological well-being [22]. Furthermore, patients after allo-HCT are at increased risks of cardiovascular events and pulmonary complications [23, 24].

Moreover, fatigue is a frequently reported adverse side-effect in cancer patients [25]. One study described the fatigue experience in allo-HCT patients during the first 100 days. In this observation, 68 % reported fatigue at the day of transplantation, 90 % at day 30 and 81 % at day 100 after allo-HCT [26]. A Cochrane review and an American College of Sports Medicine (ACSM) roundtable concluded that exercise may be an effective treatment against fatigue [2, 6]. Our recently published RCT supports these finding by demonstrating positive effects of exercise during and after allo-HCT on fatigue [13]. Mechanisms underlying the positive effect of exercise on fatigue are not fully elucidated, current models favor physiological and biological effects, for example enhanced physical performance, reduced inflammation, and less distress [27, 28].

A recent Cochrane review aimed to evaluate the effects of aerobic exercise in haematological malignancies. Exercise improved quality of life, especially physical functioning, depression and fatigue. The authors emphasised that none of the included studies investigated effects on survival. They concluded that trials with overall survival as primary endpoint are needed [29]. So far, the evidence regarding a possible influence of exercise on survival after cancer diagnosis is limited. A recent review by Ballard-Barbash et al. [30] found consistent evidence from 27 observational studies, that physical activity is associated with reduced all-cause, breast-cancer and colon-cancer specific mortality, but evidence regarding other cancers is currently insufficient [30]. Studies suggest that cardio-respiratory fitness may be a robust predictor of prognosis in non-small lung cancer patients and metastatic breast cancer [17, 31]. Courneya et al. [32] presented for the first time data from a RCT in 242 breast cancer patients, and found a non-significant trend for a better outcome (overall survival and disease-free survival) in the exercise group [32]. In addition, a small cohort study with 22 patients showed that patients with low cardiorespiratory fitness before allo-HCT had a higher risk of mortality after allo-HCT [33], furthermore, in a prospective cohort study in allo-HCT patients, the Karnofsky Performance Score (KPS) was an independent predictor of survival, and KPS <90 % was a predictor of non-relapse mortality (NRM) [34].

Data from our RCT in allo-HCT patients [13] also suggest a potential effect of exercise on survival. We observed a significantly reduced two-year total mortality (TM) for the experimental group (12 vs. 28 %, *p* = .034) after inpatient period. The effect was controlled for major confounding factors (Gratwohl Score, KPS, conditioning regime, gender, and fitness prior transplantation). Similar results were observed for non-relapse mortality (4 vs. 13 %, *p* = .017) when controlled for potential confounders. When we included the inpatient period, the risk reductions were similar but not significantly different. Interestingly, fitness at baseline was protective against NRM (*p* > .001). These results are encouraging, however, a major limitation of this study is that this was a post-hoc analysis and the study was not powered for the primary endpoint survival [35].

In consequence of the above mentioned findings, we currently perform an RCT to investigate the effects and biological mechanisms of a partly supervised yearlong exercise training on prognosis, complications, side-effects and biomarkers in patients during and after allo-HCT. To determine the specific effect of the exercise intervention itself beyond potential psychosocial effects, patients in the control group receive a comparable training schedule but with muscle relaxation according to the Jacobsen method, and the same personal attention as the exercise group.

## Methods/design

### Study design

**Fig. 1:**
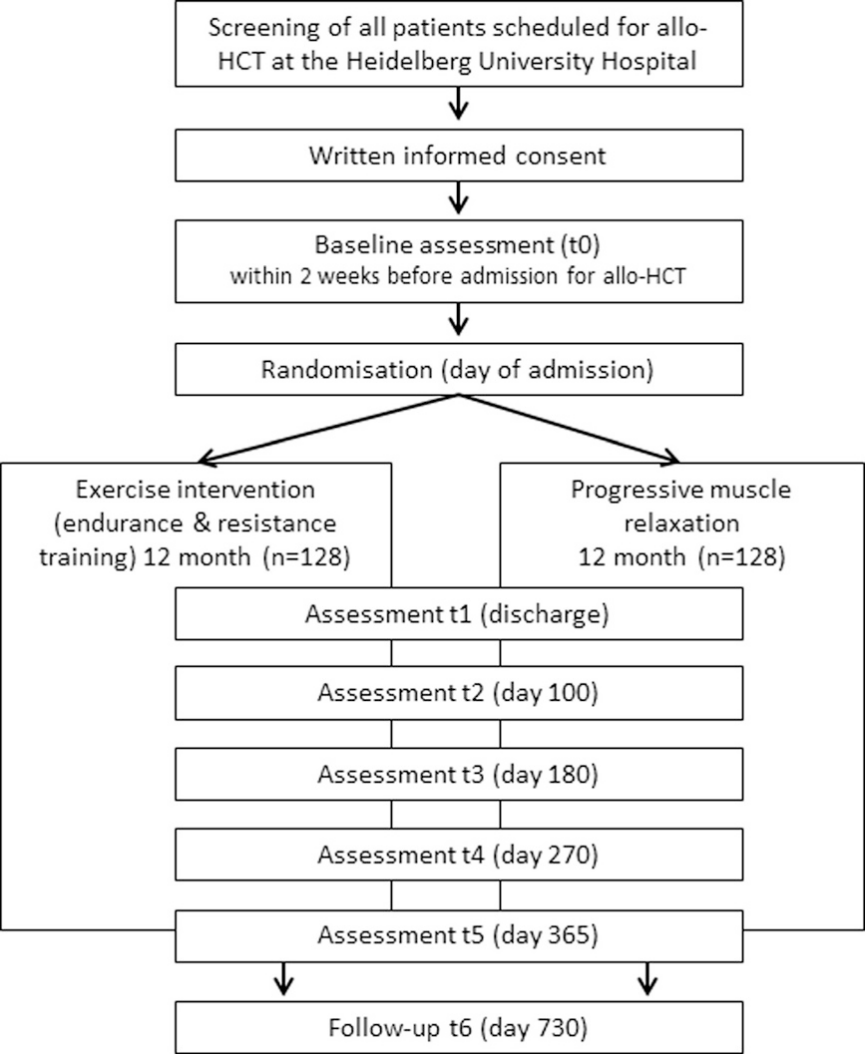
CONSORT: Study Flow of the PETRA study

The PETRA study (acronym for Physical Exercise Training versus Relaxation in Allogeneic stem cell transplantation) is a 12-month prospective, randomized, controlled clinical intervention trial in patients during and after allogeneic stem cell transplantation. Patients have to provide written informed consent prior to participation in the study. After baseline assessments, participants are randomized to a mixed type exercise program (resistance and endurance exercises) or a relaxation program over a period of one year. Both interventions are administered partly supervised. Endpoints are assessed before admission to hospital (baseline, t0), at the day of discharge (t1), day 100 after transplantation (t2), and day 180, 270 and 365 post transplantation (t3, t4, t5). Follow-up measurement will be 720 days after transplantation. [see Fig. 1].

The PETRA study has been approved by the ethic committees of the Ethic Committee II of the University of Mannheim (number 2009–349 N-MA) and Heidelberg University (number S-021/2011) and is registered at ClinicalTrials.gov (NCT01374399).

### Objectives

The primary objective of PETRA is to determine the effect of exercise on 2-year overall- survival after allo-HCT.

Secondary objectives are to estimate the effect of exercise on 2-year non-relapse mortality, median survival, fatigue, quality of life, physical fitness, including muscle strength, cardio-respiratory fitness, and body composition as well as on cognitive function. The effects of exercise on immunologic and inflammatory parameters and factors relevant for cancer prognosis and GvHD will be investigated and compared to the progressive muscle relaxation group. Finally, the sustainability of the effects will be assessed 720 days after allo-HCT.

### Outcome measures

The outcome measures used in the PETRA study are summarized in Table 1.

**Table 1:** Assessments and instruments used in the PETRA study

Outcomes	Instrument	t0	t1	t2	t3	t4	t5	t6
Primary endpoint								
Overall 2-year survival	Medical log (ongoing assessment)							
Secondary endpoints								
2-year non-relapse mortality, median (disease-free) survival	Medical log (ongoing assessment)							
Quality of life	EORTC QLQ30 / HDC-29 module	X	X	X	X	X	X	X
Fatigue	Multidimensional Fatigue Inventory (MFI)	X	X	X	X	X	X	X
Muscle strength	Isometric and isokinetic strength of representative muscle groups for upper and lower extremity measured at the IsoMed2000®	X			X		X	X
	Hand-Held-Dynamometer (isometric)	X	X	X	X	X	X	X
Cardiorespiratory fitness	CPET (VO_2_peak)	X			X		X	X
	6-min Walk Test	X	X	X	X	X	X	X
Body composition	bioelectrical impedance analysis, weight, height	X	X	X	X	X	X	X
Physical Activity	Accelerometry	X		X	X	X	X	X
Depression	CES-D	X	X	X	X	X	X	X
Distress	NCCN-Distress	X	X	X	X	X	X	X
Locus of control	KKG (German)	X	X	X	X	X	X	X
Cognitive function	Trail-making-test	X	X	X	X	X	X	X
Common side-effects	VAS, every week, later every 3 weeks							
Biomarker	Various methods (ELISA)	X	X	X	X	X	X	X
Oxidative stress marker	Analysed in urine samples	X	X	X	X	X	X	X
Others								
Socio-demographic factors	Recording of date of birth, education, occupation, familial situation, smoking, alcohol consumption	X					X	X
Medical history	Recording of pre-existing diseases, therapies	X						
Treatment data	Conditioning, complications		X					
Karnofsky Performance Score	Physician rating	X	X	X	X	X	X	X
Medication (immunosuppression, corticosteroids, analgesics)	Recorded at each visit/phone call on a medication log form	X	X	X	X	X	X	X
Physical activity history	Physical activity in adolescence, pre-diagnosis, during, and after intervention is recorded, including walking, cycling, and sports activities	X			X		X	X

### Primary outcome

Two-year overall survival data will be collected using medical logs and will be approved by the study physician (PD).

### Secondary outcomes

#### Non-relapse mortality and median survival

We will distinguish between 2-year non-relapse mortality (e.g. related to GvHD, sepsis), median survival and median disease-free survival. Survival data will be collected using medical logs and will be approved by the study physician (PD).

#### Physical fitness

*Muscle strength* is assessed by measuring isometric (4 positions) and isokinetic (1 angular velocity) maximal muscle capacity with the IsoMed 2000® diagnostic module (isokinetic evaluation and training machine). The protocol includes testing of representative muscle groups for lower (knee extensors and flexors, hip flexor and extensor) and upper (elbow flexor and extensor) extremity. Reliability and validity of isokinetic dynamometer machines have been reported in several studies [36–38]. Additionally, a hand-held-dynamometer (C.I.T. Technics) is applied to assess isometrically 6 different muscle groups within standardized test positions (knee-extensors, knee-flexors, hip-abductors, hip-flexors, elbow-flexors and elbow-extensors) [39]. Each measurement is repeated 3 times, values that are <10 % different from the median will be excluded. Hand-held–dynamometers were already applied in hematological cancer patients [13, 40, 41].

*Endurance performance* (maximum oxygen uptake, VO_2peak_) is measured by performing a symptom-limited maximal cardiopulmonary exercise test (CPET) with a step protocol (starting at 50 watts with steps of 25 watts every 2 min) on a bicycle ergometer. The criteria of exhaustion is defined as achieved estimated maximum heart rate, and respiratory exchange ratio >1.1. VO_2peak_ is defined as highest 30-s average during the test. Peak workload, peak oxygen uptake and oxygen uptake and workload at ventilatory threshold will be taken for analysis. Cardiorespiratory exercise testing is well established in cancer patients and recommendations for testing procedures as well as safety guidelines in clinical trials with cancer populations exist [42–44]. Furthermore, the six-minute walk test is applied to measure submaximal endurance performance. Heat rate and O_2_-saturation is assessed before, during and after the test and the individual perceived exhaustion is measured using the RPE-Scale [45]. For reference values the formula by Enright et al. [46] will be used. The six-minute walk test was already applied in hematological cancer patients [8, 13].

*Body composition* of the participants is estimated with bioelectrical impedance analysis (BIA). This non-invasive method determines the electrical impedance, or opposition to the flow of an electric current through body tissues to calculate an estimate of total body water, fat-free body mass and body fat [47]. BIA gives reliable measurements of body composition with minimal intra- and inter-observed variability [48]. In addition, body weight and height are measured.

*Accelerometer* (ActiGraph GT3X) is used to assess physical activity behavior. The ActiGraph is a triaxial accelerometer, that records motion in different planes and provides information about intensity, frequency and duration of physical activity [49]. Patients will wear the accelerometers during hospital stay for the transplantation and at all measurement points (except t1) for 10 days at home during daytime. Accelerometers were already used in HCT patients [50].

#### Quality of life (QoL)

QoL is assessed with the validated 30-item self-assessment questionnaire of the European Organisation for Research and Treatment of Cancer (EORTC QLQ-C30, version 3.0). It includes five multi-item functional scales (physical, role, emotional, cognitive, and social function), three multi-item symptom scales (fatigue, pain, nausea/vomiting) and six single items assessing further symptoms (dyspnea, insomnia, appetite loss, constipation, diarrhea) and financial difficulties [51]. In addition, the 29-item high-dose chemotherapy (EORTC QLQ-HDC-29) is applied, assessing common problems after stem cell transplantation, e.g. gastro-intestinal side-effects and worry/anxiety [52]. Scores will be derived according to the EORTC scoring manual. Reference values are available from the EORTC reference manual, a review, including 2,800 patients before and after HCT [53] and from a sample of the general German population stratified by gender and age [54]. Furthermore, evidence-based guidelines for the interpretation of the clinical relevance of changes in the different EORTC QLQ-C30 subscales were recently published [55], categorizing differences between scores in trivial, small, medium or large effect sizes.

#### Fatigue

Fatigue will be assessed with the Multidimensional Fatigue Inventory (MFI) which is a 20-item, multidimensional self-assessment questionnaire that has been validated for a German-speaking population [56]. It covers five different dimensions of fatigue (general fatigue, physical fatigue, reduced activity, reduced motivation and mental fatigue). Scores are derived by summing the answers (five-stage scale) of the appropriate items. Reference values of the MFI scores are available from a representative sample of the German population including 2,037 subjects [57]. The use of the MFI is recommended in cancer patients [58].

#### Distress

Distress will be assessed with the Distress-Thermometer, developed from the National Comprehensive Cancer Network (NCCN) as a screening tool [59, 60]. The Distress-Thermometer has already been validated in stem cell transplantation patients [61].

#### Depression

Depressive symptoms are assessed with the 20-item Center for Epidemiological Studies Depression Scale (CES-D). The CES-D scale is a widely used validated self-report instrument to measure current depressive symptomatology and to identify possible cases of depressive disorders, both in the general population and in patients with cancer [62].

#### Locus of control

Locus of control is measured by a validated German 21-item questionnaire *Assessment of health and sickness locus of control* (KKG). It comprises 3 subscales, assessing internal, social external and fatalistic external locus of control [63]. The questionnaire is based on the Multidimensional Health Locus of Control Scale (MHLC) [64].

#### Cognitive function

The cognitive function (concentration, cognitive flexibility) is estimated using the trail-making-test. This is a standardized, reliable and valid measure used in neuropsychological diagnostics [65, 66]. The test measures the time needed by the participant to connect numbers and letters spread over a sheet of paper in a logical sequence.

#### Biomarkers

Serum and PBMCs are derived from whole peripheral blood samples, processed within 4 h after taking the blood sample and stored at −80 °C or cryopreserved in liquid nitrogen (PBMCs) for analyses of biomarkers. Urine samples are collected for analyses of biomarkers of oxidative stress, i.e. urinary F2-isoprostane and 8-oxo-dG measured by chromatography-based methodology. Blood samples are collected for analyses of biomarkers of GvHD, i.e. inflammatory (e.g. IL-1, IL-4, IL-10, TNF-α) and endothelial parameters (e.g. angiopoetin-2, thrombomodulin (sTM)) measured by ELISA.

#### Side-effects of cancer treatment

Severity and duration of GvHD will be recorded by attending physicians every week according to the classification by Thomas et al. [67]. Furthermore, during allo-HCT and up to day 100 after, common side effects like nausea, diarrhea, appetite loss, pain, fatigue, concentration difficulties and anxiety are assessed weekly during phone calls using a visual analogue scale. After day 100 until the end of intervention (day 365 after) patients are questioned on these parameters during every phone call (depending on clinical status every 2–4 weeks). Infections are documented via medical chart review.

#### Safety issues

Potential adverse events (AEs) and serious adverse events (SAEs) causally related to the intervention or assessment procedure will be recorded. Patients are informed about contraindications for exercise sessions (thrombopenia, bleeding, infections including fever, dizziness, strong nausea/ vomiting, and strong pain) and advised to stop exercising when they feel that symptoms get stronger.

### Sample size

Sample size was chosen to achieve adequate statistical power (1-β = 0.8) for detecting a difference between the exercise and relaxation group with respect to two-year overall survival (primary outcome). The calculation is based on the two-year overall survival rates of our previous RCT (0.66 in the exercise group vs 0.49 in the control group) [35]. Accordingly, 128 participants per group (total number = 256) are needed to achieve adequate power to detect a significant (two-tailed α = 0.05) difference from Kaplan-Meier estimates tested for equality by log-rank tests.

### Participants and setting

All patients, scheduled for an allo-HCT at the Heidelberg University Clinic are invited to participate in the PETRA study. Inclusion criteria are age ≥18 years and the ability to understand and follow the study protocol. Exclusion criteria comprise contra-indications for progressive exercise training, i.e. inability to walk or stand, instable bone lesions, severe neurological deficiencies, severe cardiac or cardiovascular diseases and severe pulmonary global insufficiency.

### Recruitment and randomization

All eligible patients scheduled for allo-HCT at the University Hospital in Heidelberg are briefly informed about the PETRA study during the preparation visit (about 2–3 weeks before admission for allo-HCT) by case-management. If interested, patients are then informed in detail by the PETRA study coordinator. Upon written informed consent, the patient is included in the trial and scheduled for baseline assessment (t0), which should be within 14 days prior to start of conditioning for allo-HCT.

After completion of the baseline assessments, the participant is randomly allocated to one of the two intervention groups. Allocation is done by the minimization method [68] stratified by disease, age (< 40 / ≥ 40 years of age), gender, remission state (CR/ no CR), and intensity of conditioning (full/ doses reduced). Stratification is used in the randomization process, as we anticipate these variables to have major influence on the outcome.

### Interventions

The training starts at the same day as the conditioning treatment. Both intervention programs are performed 3–5 times per week for 12 months. During the first phase (hospital stay for allo-HCT) participants perform the program 3 times under supervision and guidance of an experienced therapist, 2 times self-directed. During the second phase (after discharge), patients perform both interventions self-directed at home. Until assessment on day 100 weekly phone calls in both groups will allow for adaptation of the program and enhance motivation/ adherence, 3 training sessions per week are recommended. After day 100 assessment, phone calls will take place every 2–3 weeks.

#### Exercise intervention

All patients receive an exercise manual with background information (including contraindications for training, motivation), instructions for tailoring the training intensity (depending on clinical status), descriptions for different resistance exercises for the whole body and endurance exercises. Patients receive stretch bands and free weights for resistance exercises, for endurance exercises patients have access to a stationary bicycle (patient room) and a treadmill (hallway) during the inpatient period. We developed a self-rating instrument which helps patients to find the appropriate exercise intensity (including RPE scale) [13]. The exercise intervention complies with the ACSM exercise guidelines for cancer survivors and healthy adults and is progressed on an individual basis [6, 69]. A complete resistance training session includes 6 to 10 exercises for major upper and lower muscle groups and is recommended 2–3 times per week. Endurance training comprises bicycling or walking/jogging 3 times per week. Patients are encouraged to increase exercise intensity when they reach 3 sets of 12 repetitions for the resistance exercises or when they feel less exhausted using the RPE-scale (target 12–14 for endurance exercise, 14–16 for resistance exercise) [70]. The exercise program is performed on a very individual basis and includes also some psychological aspects e.g. motivation, goal setting, dealing with barriers, and regular feedback on physical performance data is given to promote adherence [71]. Adherence to FITT components (frequency, intensity, type, timing) will be described [72] to ensure correct interpretation of the findings. Furthermore, it is possible to perform the training in an appropriate sport/ exercise facility close to patients’ homes. Within the scope of the PETRA study we will also develop a network called ‘*OnkoActive*’, which will enable referral to specialized exercise facilities. Furthermore, within ‘*OnkoActive*’ an internet-based training platform will be developed to support the home-based intervention.

#### Relaxation intervention

The progressive muscle relaxation method according to Jacobsen does not include any aerobic or muscle strengthening components [73]. Patients receive a manual with background information, an audio CD with 2 different versions (long/ short version) and a portable CD player. Patients in the control group will receive the standard physiotherapy program and will also have access to a bicycle ergometer and treadmill during the inpatient period.

### Data analysis

The main intervention effect will be assessed on the basis of a Cox-regression analysis between exercisers and controls (relaxation group) as defined at randomization, regardless of exercise adherence, i.e. according to the intent-to-treat principle.

The differences in secondary endpoints between groups will be assessed using mixed models, which accounts for repeated observations on the same subjects over time. This method provides a more efficient estimate of the intervention effect in pretest-post-test trials than traditional methods [74]. Mixed models will also be used for testing to which extent differences between the treatment groups depend on training adherence, changes in muscle strength, cardiorespiratory fitness, and body composition. Normality assumptions will be checked and, if deviation from normality is detected the data will be transformed accordingly.

In addition, change in physical activity behavior post intervention will be monitored on an explorative basis.

## Discussion

Treating hematological malignant diseases with allo-HCT is highly demanding for patients who experience numerous side-effects and face a very high risk of severe treatment related complications, such as GvHD and severe infections, both in the short and long-term. In general, survival rates after allo-HCT are relatively low.

Recently, our group demonstrated that there may be a positive effect of exercise on survival after allo-HCT. In our first RCT we observed a significant reduced total mortality for the experimental group (12 vs. 28 %, *p* = .034) and for NRM (4 vs. 13 %, *p* = .017). Furthermore, we observed that physical fitness prior transplantation was highly protective against NRM [35]. The cohort study by Wood et al. also suggests an association between fitness level prior transplantation and risk of mortality [33], but a small sample size of 22 patients and no use of multivariate models hamper the interpretation of these results. Against this background we designed the PETRA study. PETRA will add to current knowledge on exercise in allo-HCT patients with respect to several aspects: (1) Effect of yearlong exercise intervention on prognosis; (2) exercise effect on side-effects and complications, e.g. physical performance, QoL, fatigue; (3) exercise effect on GvHD and hematological/immunological reconstitution; and (4) sustainability and long-term effects of exercise intervention.

Given the indication of reducing mortality in cancer survivors by an appropriate physical active lifestyle, the question about possible mechanisms occurs. Some evidence suggests several potential mechanisms underlying [30]. One discussed mechanism is inflammation [75]. Several trials reported that exercise can reduce C-reactive protein (CRP), which is a marker of chronic inflammation [76–78] and change Interleukin-6 (IL-6) levels. IL-6 produced by muscle fibers during and after exercise stimulates the circulation of anti-inflammatory cytokines, such as IL-1ra and IL-10 and inhibits the production of tumor necrosis factor alpha (TNF-α) [79]. In a prospective cohort study in breast cancer patients, elevated CRP and serum amyloid A were associated with reduced overall survival [80]. Furthermore, when exercise potentially can influence parts of the immune system, the question arises, if a GvHD reaction in allo-HCT patients can also be influenced by exercise. To our knowledge, no intervention study in humans investigated a possible association between exercise and severity of the major side-effect GVHD. New results from mice experiments suggest that physical exercise can have a positive impact on GvHD. The researchers showed that endurance training (5 times per week) in mice was beneficial with regard to overall survival and also alters GvHD symptom severity. Furthermore, the authors revealed possible pathways for altering GvHD by demonstrating that exercising mice had lower levels of anti-inflammatory cytokines (IL-4 and TNF-α) [81]. Based on these findings, we aim to investigate whether exercise is able to alter GvHD severity in human adults. We are further interested to elucidate other possible exercise pathways altering GvHD by investigation of endothelial markers. Markers of interest are blood biomarkers of inflammation and TNF-α as well as endothelial markers and hepatocyte growth factor [82–85]. Natural killer cells (NK-cells) have also been shown to modulate acute GvHD, infections and recurrence [86, 87]. The positive effect of exercise on NK-cell activity in cancer patients could already be shown in studies [88], and a review about NK-cells and exercise suggests exercise as an adjunct therapy to promote expansion of NK-cell subsets [89]. Interestingly, one RCT in allo-HCT patients could already observe a positive effect of exercise during transplantation process on lymphocyte count [90].

Additionally, restoring the function of the entire haematological system after allo-HCT is an important prognostic indicator [91, 92]. Therefore, we focus also on exercise effects on haematological recovery after allo-HCT. A potential positive effect on the haematological system e.g. lymphocytes, haemoglobin could already be shown [8, 90, 93].

A central secondary endpoint is cancer-related fatigue. Fatigue is described as the most distressing side effect in cancer treatment, however, the pathophysiology of fatigue and the possible positive effect of exercise on its prevention/therapy are not well understood [94]. Therefore, our trial enables investigation of the effects of exercise on immunologic parameters as well as on biomarkers of inflammation and oxidative stress as possible mediators of fatigue [95, 96]. However, not only fatigue is a common side-effect. As a consequence of longer periods of drug intake, e.g. immune suppression and antibiotics, patients often suffer from nausea, diarrhea and further complaints. Recent studies indicated a better symptom control in exercising patients [97].

Our decision of choosing an intervention period of one year was based on the consideration that the recovery period after allo-HCT can take months to years. However, even years after allo-HCT patients have an increased risk to develop co-morbid conditions, e.g. metabolic or cardiovascular diseases [22]. On one hand, emerging research evidence indicates that these life style diseases are associated with physical inactivity. On the other hand, studies show that hematological cancer survivors are less likely to be physically active. Moreover, changing exercise behavior requires longer, individually adapted interventions. To enhance motivation and adherence, the PETRA study uses goal setting methods, and individual barriers are discussed during regular phone calls. These components have been shown to improve adherence [71]. Furthermore, PETRA helps patients to integrate in an exercise facility close to their home or give them the possibility to perform an internet-based training program. All these efforts may help patients to change the physical activity behavior in the long term. Finally, if during or at the end of the exercise intervention beneficial effects are detected, it is of interest whether those benefits sustain over a longer period of time.

Strengths of the PETRA study are the rigorous study design with a large sample size, adequately powered for the primary endpoint survival, and a broad range of assessments, including gold-standard methods for physical fitness and physical activity. Therefore, PETRA provides a unique opportunity to examine the interaction of exercise/ physical activity on the haematological system and GvHD reaction. A further strength is the choice of the control group (relaxation training). Previous exercise RCT used typically *usual care* as comparison groups. Thus, it is unclear to what extent the observed effects are based on the physical exercise effect itself, or rather on psycho-social factors related to social support or attention by the trainer. This factor is particularly important when psychosocial outcomes are measured.

## Conclusion

Previous studies in allo-HCT patients established first evidence about beneficial effects of exercise interventions on physical and psychosocial outcomes. However, the focus changed to more clinically relevant endpoints, e.g. prognosis. To our knowledge, no study has yet investigated a possible effect of exercise on overall survival after allo-HCT. Furthermore, there is a need for a better understanding of the physiological and psychological effects of exercise and their biological mechanisms in patients during and after allo-HCT. The PETRA study will provide a comprehensive picture of the potential effects of exercise during and after allo-HCT on overall survival, reducing side-effects and complications, and improving quality of life. We anticipate that our study will help to refine exercise guidelines for allo-HCT patients.

## Abbreviations

ACSM: American College of Sports Medicine
AEs: Adverse events
BIA: Bioelectrical impedance analysis
CRP: C-reactive protein
CPET: Cardiopulmonary exercise test
GvHD: Graft-versus-host disease
IL-6: Interleukin-6
KPS: Karnofsky Peformance Scale
MFI: Multidimensional Fatigue Inventory
NCCN: National Comprehensive Cancer Network
NRM: Non-relapse mortality
QoL: Quality of life
RCTs: Randomized controlled trials
SAEs: Serious adverse events
TM: Total mortality
TNF-α: Tumor necrosis factor alpha
VO2peak: Maximum oxygen uptake
